# Evolution of Cooperation with Heterogeneous Conditional Cooperators

**DOI:** 10.1038/s41598-018-22593-2

**Published:** 2018-03-14

**Authors:** Balaraju Battu, V. S. Chandrasekhar Pammi, Narayanan Srinivasan

**Affiliations:** Centre of Behavioural and Cognitive Sciences, University of Allahabad, Allahabad, India

## Abstract

Conditional cooperation declines over time if heterogeneous ideal conditional agents are involved in repeated interactions. With strict assumptions of rationality and a population consisting of ideal conditional agents who strictly follow a decision rule, cooperation is not expected. However, cooperation is commonly observed in human societies. Hence, we propose a novel evolutionary agent-based model where agents rely on social information. Each agent interacts only once either as a donor or as a receiver. In our model, the population consists of either non-ideal or ideal heterogeneous conditional agents. Their donation decisions are stochastically based on the comparison between the number of donations in the group and their conditional cooperative criterion value. Non-ideal agents occasionally cooperate even if the conditional rule of the agent is not satisfied. The stochastic decision and selection rules are controlled with decision intensity and selection intensity, respectively. The simulations show that high levels of cooperation (more than 90%) are established in the population with non-ideal agents for a particular range of parameter values. The emergence of cooperation needs non-ideal agents and a heterogeneous population. The current model differs from existing models by relying on social information and not on individual agent’s prior history of cooperation.

## Introduction

According to classical economic theory, agents are selfish rational individuals who only care about their payoffs and gains in their economic transactions. In many social interactions, agents behave like conditional cooperators who cooperate if many other agents also cooperate^1,2^. The presence of conditional cooperation has been attributed to agents’ fairness concerns and inequality aversion^2^. In this view, no ideal conditional agent would contribute to the society or cooperate because an individual agent may get a best possible payoff by free riding on others agents’ cooperation^2^. Clearly, presence of few free riders in the group leads to the decline of contributions from other agents. However, in human societies significant amount of cooperation is observed in multiple situations. Agents often donate to charities and successfully govern commons^3,4^. Many of these situations mimic conditional cooperation^3,5–8^. The non-zero contributions of agents have been attributed to their psychological aspects such as “worm glow^9^”, which does not depend on the information of other agents’ contributions and “confusion^8^”. It is also possible that agents may commit mistakes due to their bounded rationality^10^ or when they operate with noisy social information^11^.

Apart from psychology based explanations, there have been physics or game theory inspired models to explain conditional cooperation^5,11–20^. These models have considered the heterogeneous nature of population of agents and proposed stochastic rules in modelling cooperative decisions and adapting successful strategies^11,21^. It is recognized that heterogeneity of population plays an important role explaining cooperation in well-mixed and structured populations^21^. In addition, social information influences agents’ cooperative adaptive strategies^13,22^.

Models based on game theory provide a stylistic description of static situations of phenomena, but they generally ignore dynamics aspects of social learning. In human societies, agents learn or imitate successful strategies of other agents. It is important to consider social learning in strategic interactions because social learning dramatically changes the dynamic of social interactions of the agents. Further, in game theoretic models, it is assumed that agents are rational and are endowed with common knowledge (mutual knowledge) about types of agents with whom they interact. However, social information is noisy and it is hard to know about the types of agents present in social interactions. In evolutionary models of social behavior^23^, social learning is an integral part of explaining social behavior and there is no need to assume that agents are rational. It is recognized that agents are bounded rational; agents do not follow rules strictly but commit mistakes, show biases in their decisions and learn from others with certain fidelity.

The notion of bounded rationality has already revolutionized the way we understand economic decisions^24^. Recent experimental studies and theoretical models have demonstrated that bounded rationality of agents leads to cooperation when they play repeated social dilemma games^25–27^. Further some models indicate that uncertainty of social information is necessary to establish cooperation among agents^11,22,28,29^. Considering the bounded rationality of agents, we assume that agents use same stochastic rules for operationalizing their cooperative conditional decisions and imitating the successful behavior of other agents. We propose an evolutionary agent based model with heterogeneous conditional cooperators in a well-mixed population. The approach is in line with current research programs in which the psychological underpinnings of human behavior^8,24,30,31^ is combined with evolutionary approaches^9,11,23,25,26,32^ to understand interdependencies of social interactions driven by noise^11,22,29,33,34^. While human societies are structured and these structures influence population behavior, the current model does not use a structured population since there is a need to first understand the emergence of cooperation without structural constraints^13,35,36^.

We operationalize a conditional agent with its conditional cooperative criterion (*CCC*) value, whose donation decision is based on the number of other agents’ donations in the last generation or round. A conditional agent donates to another agent, if sufficient number of cooperative actions took place in the population, i.e., more than the *CCC* value of the focal agent. For instance, if an agent’s *CCC* value is *m* and there are *n* donations in the past, an ideal agent (a strict conditional agent) donates to another agent if and only if *n* > *m*, otherwise the agent does not donate. However, the agents are not always ideal conditional agents and occasionally do not follow a strict conditional rule or commit mistakes. The mistakes could be either due to noisy social information or due to bounded rationality of agents^24,30^. We consider the mistakes of agents as due to bounded rationality; even perfect social information does not prevent mistakes of agents.

The view of a conditional agent as a non-ideal (due to bounded rationality) conditional agent could dramatically change the dynamics of conditional cooperation. An ideal agent does not donate to another agent if the difference between the numbers of donations in the previous generation is less than its own *CCC* value, whereas for the same cooperative condition, a non-ideal agent can occasionally donate to the other agent. An ideal agent can operate strictly based on the conditional rule alone. The consideration of non-ideal conditional agents leads us away from a simple decision rule to a stochastic decision rule and strategy adaptation rule.

The current model with the proposed donation decision rules goes beyond the existing standard game theoretical models of conditional cooperation and is also different from the evolutionary models of human cooperation^37^ but in line with physics inspired models of conditional cooperation^11^. The standard mechanisms such as kin selection^38^, direct reciprocity^39^, indirect reciprocity^40^, spatial selection^41^, and group selection^42^ require either private or public information about the specific agents with whom they are going to interact in the future. Cooperation is achieved in these models through agents who retaliate against past defection towards them or towards others. Unlike prisoner’s dilemma, not cooperating with another agent is not the same like defection. In our model, an agent’s interaction depends on others’ actions, in general and not based on the actions of the agent with whom interaction occurs. The defection/cooperation in the conditional cooperation is based on agents’ fairness concerns rather than retaliation/reciprocation towards co-agents past actions. In the proposed model agents do not require agent specific information. The agents’ social interactions are not constrained to channelize their cooperative actions towards preferred agents.

Here, we show that depending on the degree of non-ideal nature of the agent, a population consisting of heterogeneous conditional agents interacting with each other and adapting successful agents’ *CCC* value can reach higher levels of stable cooperation. We designate the parameter that controls agent’s non-ideal nature of agent as decision intensity, *β* and the parameter that influences imitation of the successful agents’ *CCC* value as selection intensity, *η*. We show that evolution of conditional cooperation crucially depends on these parameters apart from the heterogeneity of population.

### Model

Agents interact within a well-mixed population and meet another agent randomly. At each time step, we pick two agents randomly; we randomly designate one of them as a potential donor and the other as a potential recipient. Each agent experiences several interactions in both roles but never with the same partner twice. In a given generation, we allow each agent to interact with each another agent either as a donor or as a receiver^40,43^. An agent’s payoff score is zero at birth and the score increases whenever the focal agent receives help from another agent and decreases whenever the focal agent offers help to another agent. After each generation, each agent reproduces an offspring relative to their fitness differences with randomly paired agents, simultaneously^43^.

In the beginning of a generation, each agent enters with an arbitrary *CCC* value, which is drawn from the specified uniform distribution of interval [1, *n*] where *n* ≤ *N* (*N* is population size). We assume that agents are aware of their *CCC* values and the number of cooperative actions in each generation. The non-ideal aspect of agent is modeled by using decision intensity (*β*), which controls occasional mistakes in an agent’s conditional cooperative decisions and selection intensity (*η*), which controls occasional mistakes in copying successful agents’ behavior or CCC values. Suppose, in the current generation, an agent *i* with *CCC*_i_ value donates to random met agent *j* with probability, *p*_*ij*_. The probability of donation *p*_*ij*_ is given by the equation below.

1 $${p}_{ij}=1/(1+exp(-({n}_{c}-CC{C}_{i}){\times }\beta )),$$

where *n*_*c*_ (0 ≤ *n*_*c*_ ≤ *N*) is number of donations took place in the previous generation. The higher the *β*, the agent is highly sensitive to the conditional donation rule; the agent behaves like an ideal conditional agent. For lower *β*, the agent behaves like a non-ideal agent and occasionally donates even if the conditional rule is not satisfied. For (*n*_*c*_ − *CCC*_*i*_) = 0 or *β* → 0, either an agent is confused or has lost complete information about the number cooperative interactions in the previous generation. Therefore the agent cooperates randomly by chance (*p*_*ij*_ = 0.5).

A cooperative act incurs certain cost *c* (or reduces donor fitness); in turn, the receiver gains a benefit *b* (or improves its fitness) and we assume (*b* > *c*)^44,45^. Agents accumulate fitness in their cooperative interactions. After each generation, agents reproduce offspring to the next generation with some amount of mutations^46^. We use the following reproduction process: We compare each agent with another randomly chosen agent, and giving an offspring to the one with the higher payoff. We model selection based on a pair-wise comparison process^47^.

An agent *i* reproduces its offspring to the next generation by comparing a randomly chosen agent *j*’s fitness with a probability, *q*_*ij*_

2 $${q}_{ij}=1/(1+exp(-{\rm{\Delta }}{\pi }_{ji}\times \eta )),$$

where Δπ_ji_ = (*π*_j_ − *π*_i_) is accumulated fitness difference of agent *j* and *i* in the previous generation, respectively. If *η* is high, then the high fit, medium fit, and least fit agents have 2, 1, and 0 offsprings respectively. For lower selection intensity (*η* < 1), a lower fit agent occasionally can have 2 offsprings. The process is similar to social learning with certain fidelity. An ideal agent with perfect social information copies agent *j*^th^ *CCC* value, whenever Δπ_ji_ > 0 with *η* → ∞. With *η* → 0, agent *i* does not have access to fitness information of agent *j*, or with Δπ_ji_ = 0, agent might be confused about copying the other agent’s *CCC* value. In such case, either of the two agents reproduces one offspring to the next generation. All the agents are updated simultaneously with occasional random mutations as previously mentioned. In the updating process there is no change in the population size. Next generation starts with updated *CCC* values and we set each agent payoff value to zero after each generation. The whole process is repeated after each generation for specified number of generations. We allow agents to copy a random *CCC* value, which is drawn from uniform distribution with range [1, *n*] with probability 0.1.

## Results

To understand how the degree of non-ideal nature of conditional agents helps to develop cooperation, we varied decision intensity and selection intensity and measured donation rates to quantify cooperation levels. The donation rates indicate percentage of population that cooperated in a given generation; the number of donations in a generation divided by the number of actions in the generation, and the fraction converted into percentage. Donation rates are proxy measures of amount of cooperation in population dynamics. In simulations, we observed evolution of donation rates and mean *CCC* values for 10000 generations. Simulations were performed 30 times for each experimental condition and the results were averaged to reduce individual trial variations.

In simulations, we kept population size, *N* = 100, benefit value = 1 and cost value = 0.1; these are typical parameters used in agent based models of cooperation^40^. In the initial generation, before agents start interacting with other agents, each agent’s *CCC* value was drawn from a uniform distribution [1, *n*] (*n* = *N* − 5) rather than [1, *N*]. With [1, *N*], population was dominated by agents with high *CCC* values after a few generations; hence cooperation was not established in the population for all values of *β* and *η* (*β* > 0 and *η* > 0).

We noticed that a population with homogeneous agents, in few generations, either reach high cooperation with lower *CCC* agents or reach zero cooperation with high *CCC* agents. However, cooperation is established in a heterogeneous population, with suitable *β* and *η* combinations in a gradual manner.

We observed that cooperation is not established in the population either with high *β* or with high *η*. In simulations, we arbitrarily fixed the maximum upper value of *β* and *η* to be 25. Moderate values of *β* indicate that agents behave like non-ideal agents; the agents occasionally cooperate even if the conditional rule is not satisfies and moderate values of *η* indicate that agents occasionally do not copy successful agents’ *CCC* values. Higher *β* indicates that agents cooperate if and only if the conditional rule is satisfies and high *η* indicates that the agents copy only successful agents’ *CCC* value. We tested evolution of donation rates for the parameters in the following range: 0 < *β* ≤ 25 and 0 < *η* ≤ 25; the range accounts for changes from a non-ideal conditional agent to a perfect conditional agent. In fact for *β* = 2, with (*n*_*c*_* − CCC*_*i*_) = 1, the probability of cooperation is 0.88 and probability of imitation of successful agent’s *CCC* value with *η* = 2 and △π_ji_ = 1 is the same. We observed that at the end of 10000 generations the agents’ *CCC* values were close to population mean across various experimental conditions and remained similar.

**Figure 1:**
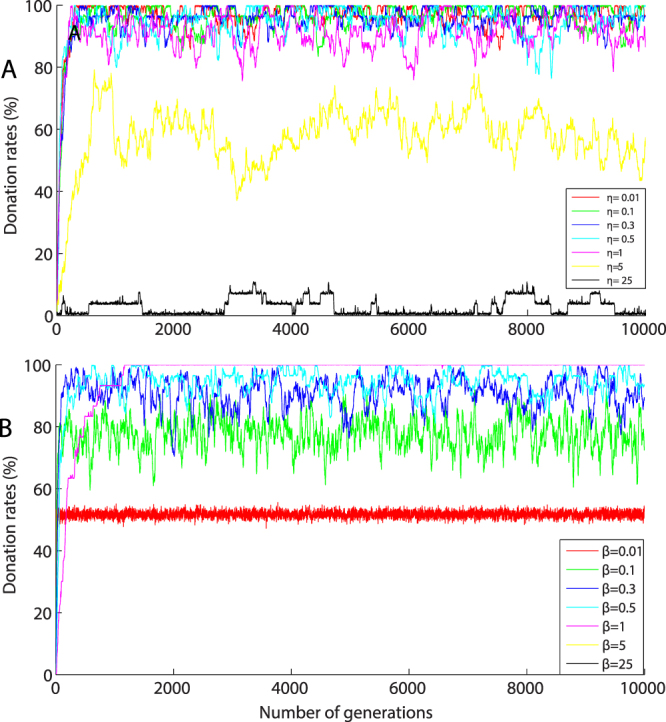
(**A**) and (**B**) depicts donation rates as a function of number of generations for particular *β* and *η* values. Each colour-coded trajectory represents the evolution of donation rates over 10,000 generations for each experimental condition with a population size of 100. (**A**) Shows that when *β* = 0.5 and 0.01 < *η* < 5 high levels of cooperation are achieved and when *η* is high, the donation rates drop to 0%. (**B**) Shows that when *η* = 0.5 and 0.01 < *η* < 2 high levels of cooperation are achieved and when *β* is high, the donation rates drop to 0%.

Figure 1 depicts evolutionary dynamics of donation rates; each color-coded trajectory represents the donation rates across the generations for fixed *β* and *η*. In Fig. 1(A) donations rates are shown for fixed decision intensity (*β*) = 0.5 with different selection intensity (*η*) values. The results show that donation rates are high and stable for lower *β* and collapses to 0% for higher *η* values. Figure 1(B) shows evolution of donation rates for fixed *η* = 0.5 with different *β* values. Donation rates are high with lower *η* and lower *β* values and the donation rates approaches 0% for higher *β* values. We note that the evolutionary trajectories are qualitatively different for changes in *β* (see Fig. 1(B)) in comparison to changes in *η* (see Figure 1(A)).

With lower *β* and lower *η* values, the donation rates increase steadily in the first few hundred generations and remain fairly stable. With either high *β* or high *η*, the cooperation levels are lower. Specifically, if the agents behave like ideal conditional agents, donation rates drop to zero.

**Figure 2:**
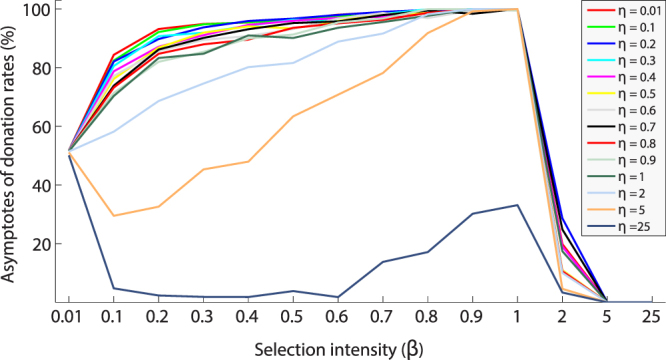
Asymptotic averaged donation rates as a function of *β* values (x-axis) and *η* values over 10000 generations. Each colour-coded trajectory represents asymptotes of donation rates for particular *β* and *η* values.

Figure 2 shows donation rate asymptotes obtained by averaging donation rates for last 25% of 10000 generations. The asymptotes provide information on steady state population dynamics. The asymptotes of donation rates steadily increases in the range of 0.01 < *η* ≤ 1and 0.01 < *β* ≤ 1. The donation rates collapse when agents behave like ideal conditional agents. The results clearly indicate that significant cooperation (more than 90% donation rate) emerges when agents behave as non-ideal conditional agents. It is interesting note that even when conditional agents’ deviate slightly from the ideal, substantial conditional cooperation is established.

We draw histograms of *CCC* values in various generations to understand composition of *CCC* values of population in the high donation rates scenarios, i.e., *β* = 0.3 and *η* = 0.1 and lower donation rates scenarios, i.e. *β* = 10 and *η* = 10.

**Figure 3:**
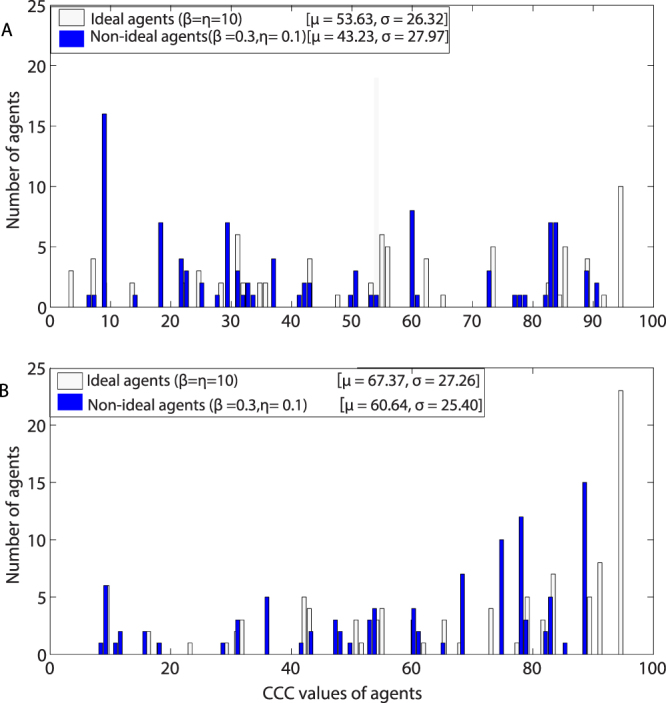
Distribution of agents based on *CCC* values in the population. (**A**) Represents distribution of *CCC* values for the 50^th^ generation and (**B**) represents distribution of *CCC* values after the 10000^th^ generation. The white bars represent *CCC* values of agents when agents are non-ideal (*β* = 0.3 and *η* = 0.1). The blue bars represent *CCC* values of when agents are ideal agents (*β* = 10 and *η* = 10). In the simulations, the population size is set to 100 and initial *CCC* values of agents were drawn from uniform distribution with range [1, 95]. The simulations were performed 30 times and the averaged results are shown in the figure.

Figure 3 depicts the distribution of agents’ *CCC* values for the 50^th^ (Fig. 3A)and the 10,000^th^ (Fig. 3B) generations with parameters *η* = 0.1and *β* = 0.3 (non-ideal conditional agents) and *η* = 10 and *β* = 10 (ideal conditional agents). The blue bars represent number of non-ideal agents and white bars represents number of ideal agents with particular *CCC* values. With non-ideal agents, high donation rates were observed; with ideal agents, zero donation rates were observed. After 50^th^ generation, there is no difference between median *CCC* values of agents; the median value for ideal agents is 49 (mean = 53.63) and median value for non-ideal agents is 48.50 (mean = 43.23). After 10,000^th^ generation the median values of* CCC* agents for ideal agents is 52 (mean = 67.37) and for non-ideal agents is 48.50 (mean = 60.64). If agents behave like non-ideal agents, the population consists of a majority of lower *CCC* value agents in the 10,000^th^ generation than if the agents behave like ideal agents. There is not much difference in the variation (standard deviation) of *CCC* values of agents in both the cases. Further, we also simulated the model by selecting initial *CCC* values of agents from normal distributions with the same mean and different standard deviations. The donation rates with a normal distribution are similar with the same mean and SD values as that of the uniform distribution. However, when we sample *CCC* values from a lesser variance normal distribution, the donation rates are less and tend to 0% when the population becomes more homogeneous.

## Discussion

Our results demonstrate that stable and high levels of cooperation are achieved in a heterogeneous population with non-ideal conditional agents. The results shows that for certain range of parameter values [*β*: (0.1 < *β* < 2) and *η*:(0.1 < *η* < 2)], high donation rates are observed in the population (see Figs 1 and 2). The non-ideal agents occasionally donate to another agent even if the conditional rule is not satisfied since the agents use a stochastic rule. The advantage of the stochastic rule is that it initiates cooperative actions whereas ideal conditional agents do not initiate cooperative actions.

In our model, when agents are non-ideal conditional agents, we observed a high level of cooperation. For instance, with *β* (0.1 < *β* < 2) and *η* (*η* < 5), more than 90% cooperation is achieved in the population (see Fig. 3). The combination of lower *β* and lower *η* allows the population maintains certain number of donation rates in each generation and these donation rates helps to achieve cooperation by non-ideal agents. However, with either very low values of *β* (*β* < 0.01) or low values of *η* (*η* < 0.01), no cooperation is observed because agents’ cooperative decisions governed by chance.

The simulations show that high donation rates emerge in a population consisting of heterogeneous non-ideal conditional agents (lower *β* and *η* values) in comparison to a population consisting of heterogeneous ideal conditional agents (high *β* and *η* values). When population consists of non-ideal conditional agents, occasional mistakes in cooperative decisions and selection always maintains non-zero cooperative actions and slightly lower *CCC* agents in the population and these agents reinforces cooperation by positive feedback loops. When population consists of ideal agents, the strict conditional cooperative decisions and selection without mistakes always drive the population towards high *CCC* value agents. A few non-cooperative actions in the population can reinforce negative feedback loops and leads to no cooperation. We observed that cooperation is stable and occasional mutations do not destroy cooperation levels when agents are non-ideal agents. For instance, few high *CCC* non-ideal agents were selected into the population by chance; these agents cooperate even if the conditional rule is not satisfied.

An ideal conditional agent’s strict condition rule might be interpreted as that agent having fairness concerns. An agent with a strict fairness concern cooperates if only if certain number of agents cooperate in the population. Clearly, cooperation decreases in a population consisting of heterogeneous ideal agents. However, cooperation can be established in a homogeneous population consisting of ideal agents^18^. It appears that non-ideal conditional agents are concerned about generosity of other agents. A non-ideal agent cooperates even if some of the agents had not cooperated in the past. The occasional violation of conditional rule might be seen by other agents as agents being generous towards the social group. Perhaps the generosity of these agents may trigger further cooperation from other agents.

It is to be noted that the notion of generosity discussed elsewhere^48^ is different from the generosity notion considered here. For instance, a generous agent using tit for tat strategy occasionally cooperates with the agent who defected in the past and the generosity is directed towards the agent who defected in the past. In the current model generosity is directed towards the social group; agents occasionally cooperate even if a number of agents have not cooperated in the past. In the former context, the agents’ action is directed towards a particular agent with whom interactions may have occurred and in the later case generosity is directed towards a social group. It seems breaking the social dilemma requires non-ideal agents and establishment of cooperation in social interactions requires heterogeneous non-ideal agents.

The simulation results indicate that only certain levels of decision intensity and selection intensity together create conditions for emergence of cooperation. Cooperation is not built in a population when the agents are strict with their conditional cooperation rule (the agents do not commit occasional mistakes in their conditional cooperation decisions, high *β*) and when agents occasionally do not copy successful agents’ *CCC* values (low *η*). While this condition creates few lower *CCC* agents, the strict conditional rule results in not creating enough number of cooperative actions. Cooperation also does not develop when agents occasionally do not follow a strict conditional rule (low *β*) and when they copy successful agents’ *CCC* values without mistakes (high *η*). In this condition, even though agents commit occasional mistakes, selection only prefers high* CCC* value agents.

The current modelling approach is inspired by stochastic rules based cooperation mechanisms proposed in physics^11^. Cooperation is established in a population when population consists of heterogeneous agents and these agents operate with stochastic cooperative rules. The internal operation of feedback loops in the model seems to provide appropriate conditions to establish cooperation^33^. The substantial number of donations observed in public good games have been attributed to agents’ confusion^8^ (confusion in understanding instructions of public good game) perhaps due to limited cognitive resources or kindness of agents^7^. These explanations mainly focused on an agent’s inability to make perfect conditional decisions; they reasoned that agents are notable to understand the instructions of game or agents are inherently endowed with altruistic preferences. The standard explanation ignores important aspects of social learning and interdependent interactions that operate in the form of nested feedback loops^49^. The mechanisms that underlie conditional cooperation described in our model potentially can help us to understand the conditional cooperation in public good games^28,29^.

The current model is implemented using a well-mixed population and no spatial structure was considered. It is well known that social structure plays an important role in explaining patterns observed in social interactions and provide natural set up for conditional strategies^12,22^. It is possible that some agents might not participate in all the repeated interactions if they obtained a poor payoff score^12^. The population might divide into groups based on their success^14^ and the identity of agents play an important role^50^. It is worthwhile to extend the current model incorporating spatial structure of populations with the above mentioned contexts.

In summary, our model provides generic explanations of conditional cooperation and can provide insight into a wide range of cooperation phenomena based on conditional cooperation. One outcome of the model is that cooperation does not develop in the population when conditional agents are ideal agents, who are strictly concerned with fairness, and population requires heterogeneity of agents. The presence of non-ideal conditional agents and heterogeneity in a population not only help in the development of cooperation but also provide stability. Perhaps evolution prefers non-ideal conditional agents than ideal conditional agents, which provide flexibility to deal with ever changing noisy social environments. In addition to contributing to the literature on conditional cooperation the model contributes to ongoing active research programs in physics and game theory^11,21^ that combine heterogeneity and stochastic nature of social interactions to understand cooperation. It is important to note that the current model does not use standard mechanisms discussed in other models^51^. In the standard models of cooperation, agents retaliate against the agent who defected with them or others in the past. Agents prefer to cooperate with agents who cooperated with them or others. The proposed model establishes cooperation based on feedback loops, which are present in complex systems and the complexity is hall mark of social interactions^33^.
